# National medicines policies – a review of the evolution and development processes

**DOI:** 10.1186/2052-3211-6-5

**Published:** 2013-07-10

**Authors:** Joëlle M Hoebert, Liset van Dijk, Aukje K Mantel-Teeuwisse, Hubert GM Leufkens, Richard O Laing

**Affiliations:** 1 Division of Pharmacoepidemiology and Clinical Pharmacology, Utrecht Institute for Pharmaceutical Sciences (UIPS), Utrecht, the Netherlands; 2 Netherlands Institute for Health Services Research (NIVEL), Utrecht, the Netherlands; 3 Department of Essential Medicines and Health Products, World Health Organization, Geneva, Switzerland

**Keywords:** National medicines policy, Medicines, Policy process, Opportunities, Development

## Abstract

**Objectives:**

Continuous provision of appropriate medicines of assured quality, in adequate quantities, and at reasonable prices is a concern for all national governments. A national medicines policy (NMP) developed in a collaborative fashion identifies strategies needed to meet these objectives and provides a comprehensive framework to develop all components of a national pharmaceutical sector. To meet the health needs of the population, there is a general need for medicine policies based on universal principles, but nevertheless adapted to the national situation. This review aims to provide a quantitative and qualitative (describing the historical development) study of the development process and evolution of NMPs.

**Methods:**

The number of NMPs and their current status has been obtained from the results of the assessment of WHO Level I indicators. The policy formulation process is examined in more detail with case studies from four countries: Sri Lanka, Australia, former Yugoslav Republic of Macedonia and South Africa.

**Results:**

The number of NMPs worldwide has increased in the last 25 years with the highest proportional increase in the last 5–10 years in high-income countries. Higher income countries seem to have more NMP implementation plans available and have updated their NMP more recently. The four case studies show that the development of a NMP is a complex process that is country specific. In addition, it demonstrates that an appropriate political window is needed for the policy to be passed (for South Africa and the FYR Macedonia, a major political event acted as a trigger for initiating the policy development). Policy-making does not stop with the official adoption of a policy but should create mechanisms for implementation and monitoring. The NMPs of the FYR Macedonia and Australia provide indicators for monitoring.

**Conclusions:**

To date, not all countries have a NMP since political pressure by national experts or non-governmental organizations is generally needed to establish a NMP. Case studies in four countries showed that the policy process is just as important as the policy document since the process must create a mechanism by which all stakeholders are brought together and a sense of collective ownership of the final policy may be achieved.

## Introduction

Medicines play a major role in protecting, maintaining and restoring people’s health. The regular provision of appropriate medicines of assured quality, in adequate quantities and at reasonable prices, is therefore a concern for all national governments [1,2]. While overuse and misuse of medicines are common in many countries, the poor availability of essential medicines is a major problem in low- and middle-income countries (LMIC) and for the poorer segments of the population [3]. In contrast to wealthier countries, up to 90% of the populations in developing countries purchase medicines with out-of-pocket payments [4,5]. Several factors contribute to increased spending on medicines across all income levels: the emergence of new diseases, population ageing, increasing antimicrobial resistance, increasing use of preventive medicines, and the availability of new and expensive medicines displaying little or no therapeutic benefit over existing treatments [6,7]. In addition to high expenditures, factors such as changing patterns of morbidity, the increasing role of the private sector in delivering medicines, health sector reforms and the presence or absence of health insurance schemes, and the effect of globalization and trade agreements also have their impact on access [8,9]. The existence of (a combination of) these factors is country specific and relates to the national political situation, as well as the economical situation and existing legislation.

These access problems have persisted despite efforts by governments, development agencies and the World Health Organization (WHO) to improve access to essential medicines, to promote rational use and to ensure that quality assured medicines are used. The reasons for the failure to achieve universal access and rational use are complex, may differ among countries, and involve a wide range of stakeholders. Thus, there is a general need for medicine policies based on universal principles, but nevertheless adapted to the national situation of a country, to meet the health needs of the inhabitants [10]. A national medicines policy (NMP) helps to identify strategies to meet these objectives, as it provides a comprehensive framework for the development of all components of the national pharmaceutical sector. A NMP typically has a future perspective of 10 years to adapt to the changing environment, and should be combined with monitoring and periodic reviews [10].

The final content of a NMP will vary among countries, as it depends on cultural and historical factors, including a country’s institutional capacity to regulate and enforce quality assurance, the political values of the government, the level of spending on pharmaceuticals, and economic development. As these factors develop continuously over time it is important to regularly update any NMP. Furthermore, the NMP must take into consideration that the elements are inter-linked and that a holistic approach is required; therefore, the development process must be clearly defined. The policy then becomes an expression of the government’s commitment to provide medicines to the population and is a framework for action [11].

Since the first publication of WHO’s ‘Guidelines for Developing National Drug Policies’ in 1988, many countries have tried to improve people’s access to essential medicines by formulating a NMP [12]. The present article reviews the historical development of NMPs in general, e.g. in terms of numbers and the status of implementation across various income levels. In addition, the policy formulation process is examined in more detail with case studies from four countries describing the historical development in these countries.

## Methods

This review is a quantitative and qualitative (describing the historical development) study of the evolution and development process of NMPs. The number of NMPs and their current status have been obtained from the results of the WHO level 1 survey 1999 (as appeared in the World Medicines Situation Report 2004), the assessment of WHO Level I indicators conducted in 2007 and the global overview of pharmaceutical sector country profiles in 2011 [13,14]. Level I indicators measure the existence and performance of key national pharmaceutical structures and processes within countries.

In the qualitative part of this study, four examples of national medicines policy formulation processes are presented: Sri Lanka (small country with a long history of pharmaceutical policy innovation), Australia (high-income Western country with an integrated policy), South Africa (large country, political struggle needed for a radical change) and the former Yugoslav Republic of Macedonia (small country with a limited capacity and affected by civil disturbance in neighboring countries). These countries were chosen because they reflect diversity in the development process of the NMP and represent a range of economic statuses. Information about these historical processes was obtained using PubMed, the 2004 World Medicines Situation report and other literature sources (year of data collection: 2009). Three experts, closely involved in the policy formulation processes in three of the four countries, were asked by email to validate the descriptions of the policy processes (July/August 2009).

## Results

### Historical development of national medicines policies

#### Role of WHO

In 1985, the Nairobi conference on ‘The Rational Use of Drugs’ took place [15]. The experts at this conference aimed to ensure access to essential medicines and rational use of medicines for all people, especially in developing countries. This meeting resulted in the recommendation that a NMP should be defined in each country to ensure that essential medicines of assured quality, safety and efficacy would be available at affordable prices to all people who need them at the right moment and at the right place and would be used appropriately. Primary responsibility for overseeing rational medicine use would rest with the individual member governments assisted by WHO. It was felt that WHO should disseminate guidelines on NMPs at the international level and this was eventually done in 1988 [1,12]. In 1989, 14 countries across the world had formulated or updated a NMP within the previous 10 years [16]. Since then, many countries have formulated a NMP.

#### Trends over time

**Figure 1:**
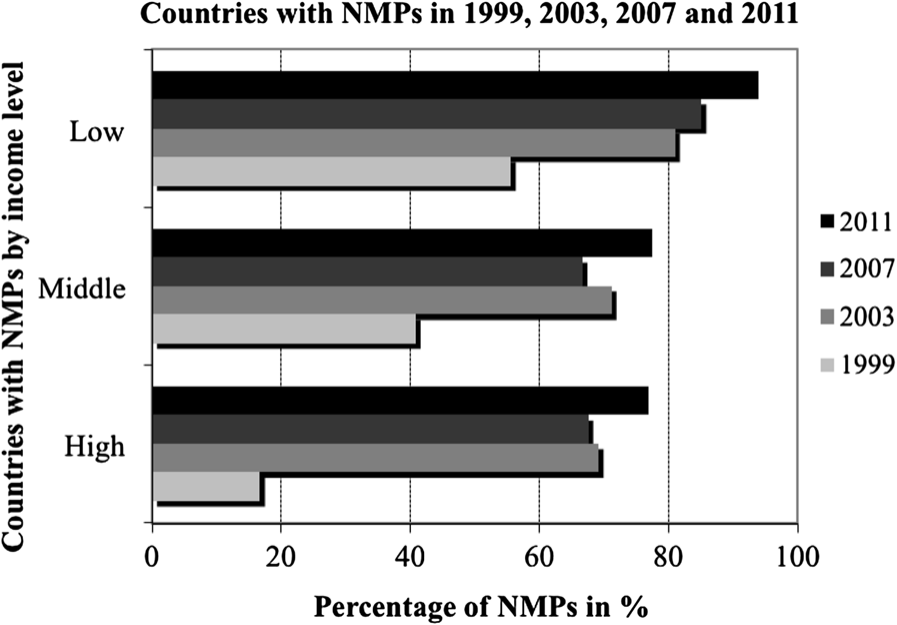
**Trends in the formulation of national medicines policies (NMP), by countries’ level of income, 1999, 2003, 2007 and 2011.** Source: WHO level 1 survey (as appeared in the World Medicines Situation Report 2004), and the global overview of pharmaceutical sector country profiles (2011). Percentages are based on number of countries surveyed by WHO.

Increased awareness of the importance of a NMP in countries with limited resources is reflected by their early development in these countries. From 1985 onwards, the number of NMPs established in low-income countries increased rapidly, with the largest increase between 1985 and 1999. In wealthier countries, formulations of NMPs began only in 1995. Figure 1 shows trends for the formulation of NMPs between 1999–2011, by income level. It reveals that the percentage of NMPs increased across all income categories but the highest proportional increase was seen in high-income countries, from 18% in 1999 to almost 80% in 2011.

#### Situation 2011

In 2011, WHO surveyed 165 countries and found that 133 (81%) countries had a NMP. A NMP implementation plan existed in 97 out of 155 (62.6%) responding countries (see Table 1). Table 1 shows that low-income countries were more likely to have a NMP compared to high-income countries. Nevertheless, these developed countries have updated their NMP more recently compared with low-income countries (data not shown).

**Table 1:** **Status of national medicines policies (NMP) by income level, 2011**[13]

		***Country income level***									
		*Low (36)*		*Lower-middle (53)*		*Upper-middle (55)*		*High (50)*		*Global Total (194)*	
		*Yes*	*%*	*Yes*	*%*	*Yes*	*%*	*Yes*	*%*	*Yes*	*%*
		*responding countries*	*Yes*	*responding countries*	*Yes*	*responding countries*	*Yes*	*responding countries*	*Yes*	*responding countries*	*Yes*
*National Health Policy exists*	Yes	30	96.8	34	87.2	23	79.3	15	83.3	102	87.2
	n	31		39		29		18		117	
*NMP exists*	Yes	31	93.9	39	86.7	33	68.8	30	76.9	133	80.6
	n	33		45		48		39		165	
*NMP implementation plan exists*	Yes	25	75.8	21	47.7	24	57.1	27	75.0	97	62.6
	n	33		44		42		36		155	

#### Development processes

A NMP is the result of a complex process of development, implementation and monitoring. First, the policy development process results in the formulation of a NMP. Secondly, strategies and activities that aim to achieve policy objectives are implemented by various stakeholders. Finally, the effect of these activities is monitored and the policy is adjusted as necessary. Throughout the process careful planning, consideration of the political dynamics and the involvement of all stakeholders is needed. Other key stakeholders besides the Ministry of Health can be found among regulators, professional organizations, producers, importers and distributors, health care professionals, patients and consumers, academics, civil society, health planners and managers, health finance authorities, insurance organizations, media and health/medicines donors, funders and major non-governmental organizations. It is important to identify political allies, and to maintain their support throughout the process [10,17].

The process of developing a NMP is almost exclusively a national matter and will differ among countries and regions with disparate income levels. In some countries, the NMP has been introduced as a complete entity (though not necessarily implemented as such), but in other countries the NMP is developed in components. In many low- and middle-income countries, a national essential medicines programme was the motivation for establishing such a policy and usually emphasized the selection, procurement, distribution and use of pharmaceuticals in the public sector [18]. During the last decade, these programmes have recognised the importance of finance tracking, medicine prices and financial management [19].

In high-income countries, the strategic goals of a NMP are generally found in various laws, regulations, and administrative procedures - rarely in a single document. The lack of an integrated national policy is unsatisfactory from a public health point of view, as some policies affecting medicines seem to contradict or undermine others [17]. Although medicines policies have generally been developed at a national level in high-income countries, international harmonization might hamper the development of a NMP within a country.

In the past two decades, market-oriented health sector reforms for meeting new health needs and requirements have been underway or under consideration throughout the world and at all income levels. Although this trend may undermine public services and pose a threat to equity in the well established social-welfare systems of high-income countries, such developments pose more immediate threats to the fragile systems in middle-income and low-income countries [20,21]. The pharmaceutical sector and its policies are influenced by health sector reforms with increased decentralisation, shifting roles and responsibilities from the central department of pharmacy management to the district level and the establishment of district pharmaceutical management points. A NMP must address the implications of an overall health system policy; there are clearly pharmaceutical policy aspects that must remain centralised such as regulation, quality assurance and public sector procurement. The process of deciding which functions fall into which area is complex and difficult and the decision to proceed, and the subsequent success of implementation, depend on political support and the capacity at a local level. Thus, the content of a NMP must be regularly monitored and adjusted if necessary.

Table 2 presents background information on population and economic data of the four countries selected for the case studies. A full description of the NMP development processes of these countries can be found in the Appendix; the most important aspects of the development processes are outlined below.

**Table 2:** General statistics of Sri Lanka, Australia, South Africa and the former Yugoslav Republic of Macedonia

	***Sri Lanka***	***Australia***	***South-Africa***	***former Yugoslav Republic of Macedonia***
*Year of policy formulation*	2006	1999	1996	2001
World Bank income level*	Lower-middle	High	Upper-middle	Upper-middle
*Region*	South East Asia	Western Pacific	Africa	Europe
Total population (million inhabitants)**	19.2	20.5	48.3	2.0
Gross national income per capita (PPP international $)**	3,730	33,940	8,900	7,850
Life expectancy at birth m/f (years)**	69/76	79/84	50/53	71/76
Total expenditure on health per capita (international $, 2006)**	213	3,122	869	623
Total expenditure on health as % of GDP (2006)**	4.2	8.7	8.6	8.2

*Income groups are classified according to World Bank estimates of 2008 Gross National Income (GNI) per capita.

**Figures are for 2006. Source: World Health Statistics 2008.

*GDP* gross domestic product, *PPP* Purchasing Power Parity, *m/f* male/female.

### Sri Lanka’s national medicines policy process: promoting generics despite opposition

The two first attempts (in 1991 and 1996) to develop a NMP failed. Two important factors in these failures were the absence of participation by civil society and the lack of a health reform campaign by civil society organizations. Thereafter, Health Action International Asia – Pacific and its network partner ‘The Peoples Movement for Rights of Patients’ began lobbying for a NMP and convened a number of national seminars, meetings and workshops on the need for a NMP, which started a development process including all stakeholders in 2005. Although accepted by consensus and endorsed by the government in 2006, the current NMP has not been implemented due to strong lobbying against the NMP by the private pharmaceutical industry even though they had participated as a stakeholder. Generic promotion and substitution are two components in the NMP that the industry vehemently opposed and they have successfully lobbied to delay the implementation of the NMP.

### Australia’s national medicines policy process: balancing health and economic objectives

Australia, as a participant at the 39th World Health Assembly in 1986, contributed to the development of the strategy calling on governments to implement a NMP. In 1991, the Australian Government established the Australian Pharmaceutical Advisory Council (APAC) and the Pharmaceutical Health And Rational use of Medicines (PHARM) Committee. APAC’s formation presented an opportunity for all interested parties to positively contribute on a multi-lateral and consensus basis to the development of the NMP, while a policy for the improvement of medicines utilisation was formulated and adopted in 1992 through the PHARM Committee and a multisectoral participatory process. In 2000 the first NMP was formally approved by the government with the overall goal ‘to meet medication and related health service needs, so that both optimal health outcomes and economic objectives are achieved’ [22,23]. To reach this goal, the policy framework addresses the inherent tensions within the objectives of attaining affordable access to medicines, while maintaining a viable pharmaceutical industry, and achieving quality medicines and health systems.

### South Africa’s national medicines policy process: focusing on equity and access

The focus of South Africa’s first single NMP was on equity. Under apartheid, the health care system was generous and highly effective, but only for the white population. Two separate draft national medicines policy documents were circulating. The key challenge for the new ANC-led government was to develop the ANC draft policy into a truly national policy, and WHO was invited to participate from the start. After one year the Minister of Health insisted that the process be completed and one policy document be prepared based on the three existing drafts (2 old drafts and a new document discussed with all stakeholders). This high-level political support resulted in the final policy document in 1996 [24]. This support also ensured that most of the national components of the policy were successfully implemented in the years that followed, although the new progressive medicine law was challenged in court by pharmaceutical industry and delayed for three years.

### Former Yugoslav republic of Macedonia’s national medicines policy process: joint effort with WHO involvement

Broad support from WHO in the FYR Macedonia, which was seriously destabilized by the Kosovo War in 1999 that led to an exodus of ethnic Albanians into Macedonia, created an opportunity to begin the NMP formulation. Prior to the enactment of the Health Care Law in 1991 and the establishment of the Ministry of Health, the system of health care was fragmented with little central governance or strategic overview although it offered universal accessibility [25,26]. In February 2000, the Ministry of Health and the WHO Humanitarian Assistance Office in Skopje organized an initial meeting to discuss the implementation of a NMP and to present the main aspects of a NMP. In May 2001, several drafts were combined to produce one comprehensive document which was officially endorsed by the FYR Macedonian government in October 2001.

## Discussion

The number of NMPs around the world has increased over the past 25 years with an early increase in low-income countries and a more recent increase in high-income countries. Nevertheless, to date, not all countries have a NMP. If there is no political pressure by national experts or non-governmental organizations the need to establish a single comprehensive document may be absent. Low-income and lower middle-income countries may be more likely to have a NMP, because access to medicines is a challenging problem for politicians. In addition, WHO has focused on low- and middle-income countries since the 1980s. In high-income countries access is generally assured, but complex issues related to rational use, medicines prices, reimbursement and industry concerns confront policy makers. Although most wealthier high-income countries have managed without a comprehensive NMP, they sometimes encounter problems due to a lack of a single NMP [17]. In most high-income countries components of a medicines policy are often in place, but are rarely addressed in a single comprehensive national medicines policy. In the USA, for example, matters tend to be managed separately by the Food and Drug Administration (FDA; regulation), the Federal Trade Commission (FTC; trading and competition issues), the National Institutes of Health (NIH; research) and by individual states (dispensing). Moreover, states and professional associations develop and implement many different aspects of a medicines policy.

The policy development processes of the four case studies show that the development of a NMP is a complex process that is country specific. Lessons learnt from the four described policy processes demonstrate that an appropriate political window is needed for the policy to be passed; for South Africa and the FYR Macedonia, a major political event acted as a trigger for initiating the policy development. Furthermore, all stakeholders must be involved at an early stage to offer a stable system that guarantees access, and rational use of medicines. During the policy development process, countries are forced to develop a transparent framework so that stakeholders understand their roles and responsibilities. Countries are also forced define national priorities based on a balance between meeting patients’ needs as well as ensuring effective use of the countries’ resources and other incentives (e.g., maintaining a viable national industry as seen in the Australian case study).

Policy-making, however, does not stop with the official adoption of a policy but should create mechanisms for implementation and monitoring. Large differences exist between NMPs in how the implementation is managed and funded. Unless there is a performance-related budget linked to the policy, adequate implementation (and monitoring) is unlikely to occur. The policy process of Sri Lanka clearly showed the struggle to implement the policy due to generics use guidelines, which the local industry opposed. A clear policy should be reassessed from time to time and revised as appropriate - ideally every 4–5 years. Sufficient staff with appropriate technical and professional capabilities is required [27]. Indicators or performance standards are a tool to determine whether adequate progress is being achieved and to assess the effects of changes in medicines policy objectives. Independent consultants or external professionals may be invited to complement a national evaluation team. Resources needed for these revisions should be allocated from the start of the development process. NMPs may address the importance of monitoring and evaluation, but indicators for monitoring or an actual monitoring framework are often lacking within the policy. For the selected countries, the NMPs of the FYR Macedonia and Australia provide indicators for monitoring. None of the policies included independent external evaluation of the implementation of the NMP.

Even if a NMP indeed exists in a Ministerial Declaration or even in the law, and an implementation plan exists as well, this does not always mean that the policy works effectively. Shortcomings in regulatory performance, lack of access to essential medicines and irrational use may exist despite the existence of a comprehensive policy document. An example of this shortcoming was seen in the failure to protect Australia (and other countries) from the COX-2 inhibitor regulatory failure [28]. Thus, a more complex NMP with major divisions of responsibilities between the central and state governments must include both the easily agreed-upon common interests but must also resolve the conflict areas in order to reach an agreed, national compromise involving all parties.

Although most pharmaceutical problems are best addressed at the national level through the use of NMPs, there could be cases where medicines policy issues are better managed at a regional or global level because some problems extend beyond the boundaries of national borders. There are many regional organizations working together to harmonise the regulatory aspects of NMPs, e.g. the European Medicines Agency (EMA) centralised registration process, and the implementation of various aspects of the NMP. Regional groupings such as ASEAN and COMESA are collaborating to harmonise regulation and pricing information and coordinate NMPs across countries within their regions.

## Conclusion

Experiences in many countries have shown that pharmaceutical problems can best be addressed in a comprehensive policy, as piecemeal approaches can leave important problems unsolved. Case studies in four countries showed that the policy process is just as important as the policy document since the process must create a mechanism by which all stakeholders are brought together and a sense of collective ownership of the final policy may be achieved. This may be crucial in view of the challenges to implement and monitor the NMP.

## Appendix

### Full description of NMP development policy processes

#### *Sri Lanka’s national medicines policy process: promoting generics despite opposition*

In 1959, Sri Lanka had a limited list of essential medicines and the use of generics was compulsory in public health care. Professor Senaka Bibile, a Sri Lankan pharmacologist, played a leading role in developing a rational pharmaceutical policy which ensured that the poor people would get good quality medicines at the lowest possible price to the country and that doctors would prescribe the minimum required medicines to treat the patient's illness. The country’s limited list of medicines was extended to the private sector in 1972, when the state became the sole importer of all pharmaceuticals through its trading arm, the State Pharmaceuticals Corporation (set up by professor Senaka Bibile), which also supplied the private market. By setting up the State Pharmaceutical Corporation and calling for worldwide bulk tenders the stranglehold of multinationals on the medicines trade was broken. This made them compete with each other and with generic medicines producers which enabled poor countries to obtain medicines much cheaper. This policy was supported by WHO and other UN agencies with enormous benefit to developing countries. [28] The government’s attempt to extend control to the private sector provoked controversy within the health services and the private sector, and particularly within the pharmaceutical industry. Cooperation with international organisations and non-governmental organisations was necessary to develop and implement a medicine policy that addressed the country’s growing dependence on a number of multinational companies that monopolised the global trade in medicines [29]. In 1977, a new government came into power with neoliberal policies. The limited list of medicines was applied only to the public sector and the use of brand names and aggressive promotion of brands in the private sector returned. In 1991 an attempt was made to develop a NMP, but the attempt failed as did a subsequent attempt in 1996. These efforts were not confined to the NMP alone; both in 1991 and 1996 a health task force was set up to recommend ways and means of restructuring the entire health service system. The documents they produced were accepted by the Ministry of Health, but were not endorsed by the government. Two important factors for this failure were the absence of participation of civil society in the two task forces and the lack of health reform campaigns by civil society organizations.

In 2006 Sri Lanka succeeded in developing a NMP. In that year, the process was quite different from the previous attempts. Health Action International Asia – Pacific (HAIAP) and its network partner ‘The Peoples Movement for Rights of Patients’ (PMRP) began campaigning and lobbying for the formulation of a NMP and convened a number of national seminars, meetings and workshops on the need for a NMP. In 2005, two workshops facilitated by WHO/SEARO were held for all stakeholders, including representatives from the Ministry of Health, academia, health professionals and associations, trade unions, the private pharmaceutical industry and trade, and civil society. HAIAP and PMRP took an active role and the NMP was accepted by consensus, forwarded to the Ministry of Health, approved by the cabinet, and passed by the Parliament in 2006.

The objectives of the current 2006 NMP for Sri Lanka for both public and private sectors are:

1) to ensure the availability and affordability of effective, safe and quality medicines relevant to health care needs of the people in a sustainable and equitable manner;

2) to promote the rational use of medicines by healthcare professionals and consumers;

3) to promote local manufacture of essential medicines [28].

In the three years since the endorsement of the policy by the government, the Ministry of Health appointed a National Standing Committee (NSC) with 18 members representing all stakeholders and a mandate to implement the NMP. The NSC appointed a subcommittee to prepare a draft ‘Act to Regulate Medicinal Drugs and Devices, Cosmetics, Neutracentical and Functional Foods’. The draft was presented to the Ministry of Health in early 2008; however, little has happened to the data of data collection (2009). There was strong lobbying by the private pharmaceutical industry and trade against the NMP even though they had participated as a stakeholder. Generic promotion and substitution are two components in the NMP that the industry vehemently opposed and they have successfully lobbied to delay the implementation of the NMP. The PMRP has filed a fundamental rights petition in the supreme court of Sri Lanka asking the court to direct the Ministry of Health to implement the NMP. PMRP argues that any delay in the implementation causes a denial of the fundamental right of the people to access life saving medicines at affordable prices (Balasubramaniam K. Personal communication).

#### *Australia’s national medicines policy process: balancing health and economic objectives*

Australia, as a participant at the 39th World Health Assembly in 1986, contributed to the development of the strategy calling on governments to implement a NMP. The need for a comprehensive NMP was further illustrated in the ‘Health for All Australians’ document issued jointly by all State and Federal Australian Health Ministers in 1988. It was recognised that there was considerable medicine-related morbidity and mortality in Australia, much of which was preventable. There were, however, very few strategies or (inclusive) structures in place to support improvements in medication use. Furthermore, the research efforts and knowledge of successful strategies to improve medication use were also limited, both within Australia and internationally. In 1989, the Consumers Health Forum widely circulated a document ‘Towards a National Drug Policy’ which crystallised the concept of an integrated medicine policy (combining several current existing ad-hoc policy measures) and the need for action on how medicines are used. In 1991, the Australian Government established the Australian Pharmaceutical Advisory Council (APAC) and the Pharmaceutical Health And Rational use of Medicines (PHARM) Committee. Following the successful establishment of PHARM and APAC, the government had begun to consider the articulation of a National Medical Drug Policy. A policy for the improvement of medicines utilisation was formulated and adopted in 1992 through the PHARM Committee and a multisectoral participatory process.

Eight years later, in 2000, the first NMP was formally approved by the Australian government [22,23]. Under the auspices of the APAC, the policy integrated pre-existing elements within the new Quality Use of Medicines policy. The NMP was formulated in a partnership of government, healthcare professional organisations, the pharmaceutical industry, distributors, healthcare consumers and other stakeholders. APAC’s formation in 1991 presented an opportunity for all interested parties to positively contribute on a multi-lateral and consensus basis to the development of the NMP. Australia is one of the few developed countries with a comprehensive NMP and the most recent example of a high-income country establishing a NMP. The Australian policy’s four major objectives are to ensure:

1) timely access to the medicines that Australians need, at a cost individuals and the community can afford, through the Therapeutic Goods Administration and through the Pharmaceutical and Repatriation Benefits Schemes;

2) that medicines meet appropriate standards of quality, safety and efficacy;

3) maintaining a responsible and viable national pharmaceutical industry, through the industry portfolio;

4) quality use of medicines [23].

The overall policy goal of Australia’s NMP is ‘to meet medication and related health service needs, so that both optimal health outcomes and economic objectives are achieved’. To reach this goal, the policy framework addresses inherent tensions within the objectives of attaining affordable access to medicines while maintaining a viable pharmaceutical industry, and achieving quality medicines and health systems.

Political pressure by national experts and non-governmental organizations had a major influence on the development of Australia’s NMP [11,30-32]. While much has been achieved in a decade, the development and marketing of new medicines, the use of new technologies and sources of medicines information, the costs of medicines, and perhaps most importantly, the increased interest consumers have taken in their health care, present further issues for policy development and implementation [33].

NMPs often do not address problems in other countries or what has been experienced when safety issues were at stake. Although this is a matter that concerns many countries, Vitry and colleagues showed that policy stakeholders failed to protect Australia from the COX-2 (cyclo-oxygenase-2) inhibitor regulatory failure, despite the fact that Australia’s NMP aims to ensure quality use of medicines. They found that regulators did not appropriately warn prescribers about potential cardiovascular risks. The Pharmaceutical Benefits Scheme (PBS) did not limit unjustified expenditures on COX-2 inhibitors and pharmaceutical companies ran intense and misleading promotional campaigns on COX-2 inhibitors without adequate controls. Independent medicines information was insufficient to counter the effects of the millions of dollars spent on advertising in Australia. Their conclusion was that the core elements of the NMP, in particular the medicine approval process, the post-marketing surveillance system, the control of medicine promotion, and the quality of independent medicine information, required major reappraisal to avoid similar disasters in the future [34]. Fundamental to this is the development and utilization of performance indicators to provide a set of objective criteria by which the implementation and effect of strategies for quality use of medicines can be monitored [35,36].

#### *South Africa’s national medicines policy process: focusing on equity and access*

In 1993, prior to the first democratic elections after apartheid, two separate draft national medicines policy documents were circulating. One was written by the government at the time, with input from academies at the University of Cape Town (a famous ‘white’ university), and another by the African National Congress (ANC). After a democratically elected ANC-led government was established in April 1994 under President Mandela, a national pharmaceutical policy committee was appointed by the Minister of Health with the following objectives:

1) develop a pricing plan for medicines to be used in South Africa in the public and private sectors;

2) develop a plan to ensure that medicines are tested and evaluated for effectiveness in the South African context of treatment using epidemiological approaches;

3) develop an Essential Medicines List to be used in the public sector and prepare treatment guidelines for health personnel;

4) develop specific strategies to increase the use of generic medicines in South Africa;

5) prepare a plan for effective procurement and distribution of medicines in South Africa, particularly in the rural areas;

6) investigate traditional medicines; and

7) rationalize the structure for pharmaceutical services [24].

The key challenge for the new government was to develop the ANC draft policy into a truly national policy, and WHO was invited to participate from the start. In November 1994, the committee presented a first report of its findings to the Minister of Health, and a new discussion document was disseminated based on the recommendations. This draft was used as the basis for wide consultations and discussions with health care providers, academia, other ministries, provincial and district representatives, professional organizations, pharmaceutical industry and patients. The process took time and after one year the Minister of Health insisted that the process be completed and one policy document be prepared based on the three existing drafts. This high-level political support resulted in the final policy document which was adapted by the Cabinet and published in 1996 [24].

The focus of the new policy was on equity. Under apartheid, the health care system was generous and highly effective, but only for the white population. Less than one quarter of the national health care budget was left for the remaining three quarters of the population along legally defined racial categories. The real challenge was to reduce overconsumption in the sophisticated parts of the system, e.g. the teaching hospitals, without losing their good quality and reputation, and make these facilities available for everyone. Another challenge was to use the savings to strengthen the rural services, which were the main source of care for the majority of the population. In all segments of the system, overuse and waste of medicines had to be reduced. Key tools, in this respect, were the development of national treatment guidelines and lists of essential medicines for all levels of health care. WHO contributed to the policy process by breaking the technical isolation that international sanctions had caused, and by supplying the government with information on practical experiences from successful countries, such as Zimbabwe and Australia. WHO also acted as an ‘honest broker’ to support the government to reach consensus among the various stakeholders, and helped develop and implement a five-year technical support programme - the South Africa Drug Action Programme (SADAP) [37].

The policy was largely successful, especially due to the political window of opportunity after the 1994 election and the high-level political support. Most of the national components of the policy (treatment guidelines, national medicine list, review of the national regulatory agency) were successfully implemented in the years that followed. However, the new medicine law, which included several progressive, but controversial, pricing policy components, such as generic substitution and parallel importation, was challenged in court by the research-based local and international pharmaceutical industry and delayed for three years. For various political reasons, some of the provinces remained skeptical and hesitated to become partners in the process. Yet, overall the policy was effective in making the government and the various stakeholders aware of the need for change, and in paving the way for the development of the pharmaceutical sector in the decade to follow. Several senior government officials, now in high office, were involved in SADAP in their formative years. The policy document of 1996 remains a strong text which can serve as an example for other countries [10,38].

#### Former Yugoslav Republic of Macedonia’s national medicines policy process: joint effort with WHO involvement

The Former Yugoslav Republic (FYR) of Macedonia became independent in 1991 following the break-up of Yugoslavia and remained at peace during the Yugoslav wars of the early 1990s. However, the country was seriously destabilized by the Kosovo War in 1999. Following the exodus of ethnic Albanians from Kosovo into Macedonia and Albania at the end of the Balkan civil conflict, broad support from WHO in the FYR Macedonia also created an opportunity to begin NMP formulation. Prior to the enactment of the Health Care Law in 1991 and the establishment of the Ministry of Health, the system of health care was fragmented with little central governance or strategic overview, although it offered universal accessibility [25,26]. In February 2000, the Ministry of Health and the WHO Humanitarian Assistance Office in Skopje organized an initial meeting to discuss the implementation of a NMP and to present the main aspects of a NMP. In April 2000, a group of 14 experts were appointed by the Minister of Health to work on the development and formulation of the NMP strategy document. Five working groups were created to develop specific elements of the policy: legislation and regulations, medicine selection, medicine information, rational medicine use, supply and economic strategies, and human resource development. Several meetings which were facilitated by international consultants were held before the NMP adoption workshop. In May 2001, several drafts were combined to produce one comprehensive document which was officially endorsed by the FYR Macedonian government in October 2001. While the Ministry and the Health Insurance Fund have continued to further develop and implement the medicines policy, a national working group completed an analysis of the pharmaceutical sector under the umbrella of the WHO Good Governance for Medicines Project [39]. In collaboration with the Ministry of Health, the policy implementation will be supported in the future. The country’s NMP includes a list of indicators for monitoring NMP implementation [40,41].

## Competing interests

The division of Pharmacoepidemiology and Clinical Pharmacology, Utrecht Institute for Pharmaceutical Sciences, employing authors JH, AM-T and HL, has received unrestricted research funding from the Netherlands Organisation for Health Research and Development (ZonMW), the Dutch Health Care Insurance Board (CVZ), the Royal Dutch Pharmacists Association (KNMP), the private-public funded Top Institute Pharma (http://www.tipharma.nl, includes co-funding from universities, government, and industry), the EU Innovative Medicines Initiative (IMI), EU 7th Framework Program (FP7), the Dutch Medicines Evaluation Board, the Dutch Ministry of Health and industry (including GlaxoSmithKline, Pfizer, and others). LvD has received unrestricted grants from GlaxoSmithKline (for a study on patient empowerment) and Bristol-Myers Squibb and Astra Zeneca (for a study on the consequences of diabetes for work). RL declares not to have a competing interest.

## Authors' contributions

JH was responsible for the conduct of the study and for the drafting and revising of the article. ROL had the original idea for the article and participated in the collection of additional literature and contributed to the writing of this manuscript. LvD, AMT and HL supervised study conduct and drafting of the manuscript. All authors read and approved the final manuscript.
